# Dendrimers as Modifiers of Inorganic Nanoparticles for Therapeutic Delivery in Cancer

**DOI:** 10.3390/pharmaceutics15020398

**Published:** 2023-01-24

**Authors:** Mkhuseli Zenze, Aliscia Daniels, Moganavelli Singh

**Affiliations:** Nano-Gene and Drug Delivery Group, Discipline of Biochemistry, University of KwaZulu-Natal, Private Bag X54001, Durban 4000, South Africa

**Keywords:** dendrimers, nanoparticles, dual delivery, cancer, therapeutics

## Abstract

The formulation of nanoscale systems with well-defined sizes and shapes is of great interest in applications such as drug and gene delivery, diagnostics and imaging. Dendrimers are polymers that have attracted interest due to their size, shape, branching length, amine density, and surface functionalities. These unique characteristics of dendrimers set them apart from other polymers, their ability to modify nanoparticles (NPs) for biomedical applications. Dendrimers are spherical with multiple layers over their central core, each representing a generation. Their amphiphilic nature and hollow structure allow for the incorporation of multiple drugs or genes, in addition to enabling easy surface modification with cellular receptor-targeting moieties to ensure site-specific delivery of therapeutics. Dendrimers are employed in chemotherapeutic applications for the delivery of anticancer drugs. There are many inorganic NPs currently being investigated for cancer therapy, each with their own unique biological, chemical, and physical properties. To favor biomedical applications, inorganic NPs require suitable polymers to ensure stability, biodegradability and target specificity. The success of dendrimers is dependent on their unique structure, good bioavailability and stability. In this review, we describe the properties of dendrimers and their use as modifiers of inorganic NPs for enhanced therapeutic delivery. Herein, we review the significant developments in this area from 2015 to 2022. Databases including Web of Science, Scopus, Google Scholar, Science Direct, BioMed Central (BMC), and PubMed were searched for articles using dendrimers, inorganic nanoparticles and cancer as keywords.

## 1. Introduction

Nanotechnology has had a significant impact on various areas of scientific research including improvement of healthcare. Considering its multidisciplinary applications (chemistry, medicine, engineering, electronics, optics, and biomaterial science), the design and use of nanotechnology in these fields have grown exponentially in recent years, with biomedical applications being extended to early diagnosis and imaging of diseases such as cancer. Patients diagnosed with breast, lung, colon, prostate, and ovarian cancer often have occult or visible metastatic colonies. With the emergence of diagnostic nanotechnology, these numbers are expected to decrease significantly [1].

Cancer remains one of the leading causes of death globally. According to a study by the American Cancer Society (ACS), approximately 1.9 million new cases of cancer were diagnosed, and 609,360 deaths cases were reported in 2022. The current therapeutic strategies being implemented for different types of cancers include surgery, chemotherapy, and radiation therapy or, in some cases, a combination of two such strategies [2]. Chemotherapy drugs are generally not fully effective due to their limitations, which include poor solubility, short blood circulation time, and lack of selectivity for normal and cancer cells that can cause serious side effects and lower survival rates [3]. Scientific efforts have been made to develop nanocarriers that efficiently deliver therapeutics to the tumor site while reducing side effects and increasing specificity. Various biomaterials such as lipids, polymers, inorganic nanoparticles (NPs) and carbon-based NPs are being investigated as potential therapeutic carriers [4].

The development of NPs as drug delivery systems and as drugs themselves has had a significant impact on drug delivery [5]. In biomedical applications, the design and engineering of NPs with well-defined particle sizes and suitable shapes are of utmost significance, especially for gene/drug delivery, imaging and photothermal therapy (PTT). Due to their physicochemical properties and high thermal conversion rate, most inorganic NPs have attracted interest for PTT, which is non-invasive and can be tumor-specific. When used in combination with gene or drug delivery, PTT can improve therapeutic efficacy [6]. To improve their ability to traverse cellular membranes and lower the risk of unwanted clearance from the body through the liver or spleen, these NPs should be designed at optimized sizes < 200 nm and with a uniform distribution, as NPs >200 nm can be easily removed by the reticuloendothelial system [7,8].

Among the array of NPs available, organic and inorganic NPs feature most prominently. Inorganic NPs have been extensively used in a wide range of biomedical applications, including magnetic resonance imaging (MRI) [1,2,3,4], drug release [9,10], gene transfer [11,12], biosensing [13,14], immobilization of proteins and enzymes [15,16], various cancer treatments, and cell separation [17,18]. It is essential to develop tunable multifunctional inorganic NPs with various surface modifications to enhance their physicochemical characteristics, such as biocompatibility, stability, and solubility under physiological conditions.

Dendrimers/dendrons [19,20], polyethylene glycol (PEG) [21,22], polyvinyl pyrrolidone (PVP) [22,23], poly-L-lysine [24], and chitosan [22] are a few prominent natural or synthetic polymers that have been intensively studied as favorable surface coatings for various types of inorganic NPs in recent years. Dendrimers have been actively researched as NPs and as modifiers for NPs, adding a new dimension for cancer research with an array of potential treatments. Dendrimers have well-defined surface groups that can be conjugated to a variety of biomolecules, including antibodies, aptamers, nucleic acids, targeting ligands, imaging probes, drugs, and biosensing agents [25]. Dendrimers can be used as surface-functionalizing or coating agents in dendrimer-based inorganic NPs for various biomedical applications [26]. In this review, we look at recent developments in the biomedical applications of dendrimers as modifiers of inorganic NPs. To date, there has been a limited number of reviews on the use of dendrimers as inorganic NP modifiers. In this paper, we place particular emphasis on the poly(amidoamine) (PAMAM) dendrimers, which are the most thoroughly researched family of dendrimers. The current review is not limited to the use of dendrimers in biomedicine; we also strive to provide information on the physiological and toxicological properties of dendrimers, in addition to highlighting advantages in the application of dendrimers as modifiers of inorganic NPs for cancer therapy. In this review, we not only showcase their versatility in nanomedicine but also suggest new possibilities for research to enhance their therapeutic use.

## 2. PAMAM Dendrimers 

**Figure 1:**
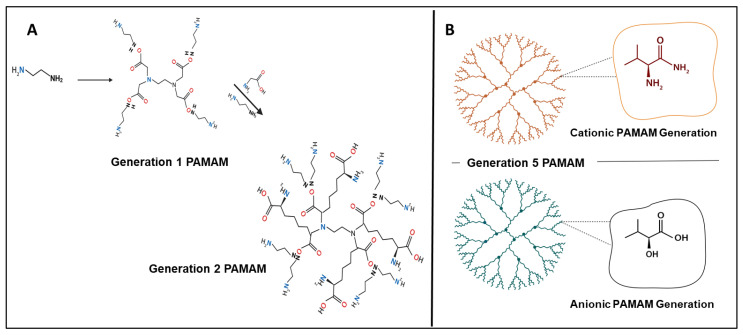
Schematic presentation of PAMAM dendrimer: (**A**) showing synthesis from G-0 to G-2, and (**B**) presenting G-5 PAMAM with cationic and anionic structures (Created with BioRender.com, accessed 6 December 2022).

The first full nanosized family of dendrimer structures to be chemically synthesized, characterized, and commercialized is polyamidoamine (PAMAM) dendrimers. PAMAM dendrimers are typically spheroidal, highly branched cascade polymers, and during synthesis, their size, surface functional groups, and charge can all be precisely regulated. Divergent step-growth polymerization in a layer-by-layer fashion (expressed in “generations” or “G”), usually centered around an initiator two-carbon ethylenediamine core unit, though ammonia and cystamine, can be utilized to create PAMAM dendrimers. As a result, successive generations of dendrimers (Figure 1A) with defined molecular structure, branching points, terminal functional chemistry, and very low polydispersity are produced by layers of radially repeating units coupled to the core [25,26,27]. With twice as many terminal surface groups as its immediate precursor, each subsequent generation results in an increased diameter and molecular weight (Figure 1B). In addition to the conventional cationic PAMAMs with their amino-terminal surface chemistry (-NH_2_), hydroxyl- (-OH, neutral), and carboxyl- (-COOH, anionic) groups have been recently synthesized and are commercially available [28]. Anionic PAMAMs are synthesized in half generations, in contrast to cationic and neutral PAMAMs, which are both available as full-generation dendrimers (G1 through G10, e.g., G1.5 to G9.5). PAMAM dendrimers are thus offered as a homologous series of polymer structures with increasing molecular weight (or generation) and various surface chemistries that provide a defined set of physiochemical properties to assess structure–activity relationships in drug delivery, as well as in biological functions [29]. Another benefit is that PAMAM dendrimers can be modified post synthesis to create different structural designs. For instance, “activated” dendrimer structures with reduced internal structural branching and increased internal cavity capacity can be generated using a controlled solvolytic technique at high temperatures. Such PAMAM nanocarriers enable improved drug entrapment and delivery by increasing the amount of drug payload that can physically be adsorbed internally within the cavities of the dendrimer [30]. The sixth-generation (G6) PAMAM dendrimer called SuperFect is an example of a commercially available terminal amino-functionalized dendrimer. PAMAM dendrimers are therefore thought to be perfectly suited to act as non-viral delivery vectors, allowing the therapeutic cargo to be carried and protected either within the internal cavities (Figure 1A), bound to the surface groups, or by a combination of these two approaches, which may be useful for drug combination therapies [31].

In order to increase solubility, decrease toxicity, and perhaps even conceal taste while also increasing the pharmacokinetics and bioavailability of the active compound, drug molecules are often trapped inside the dendrimer cavity [32]. Compared to later generations (>G6), which have more stiff surfaces because of high branching and surface group density, activated or earlier generation PAMAMs (usually below G4) with open structures have a greater capacity for drug entrapment [33]. Furthermore, covalent conjugation or electrostatic adsorption can be used to bind molecules or bioactive components to the dendrimer surface. Complexes formed from PAMAM dendrimers with nucleic acids are commonly referred to as “polyplexes” or “dendriplexes”, which can be used efficiently to deliver nucleic acids across the cellular membrane. Cationic amino-terminated PAMAM G5 dendrimers have been reported as inorganic NP modifiers in gene delivery systems for nucleic-acid-based therapies, including plasmid DNA [34,35], messenger RNA (mRNA) [36], antisense oligonucleotides and small interfering RNA (siRNA) [37,38]. Furthermore, cationic G6 PAMAM dendrimers have been shown to be able to modulate the signaling of mitogen-activated protein kinases (MAPKs) in vivo [39,40].

PAMAMs, in addition to their ability to improve drug/gene delivery, should ideally be physiologically inert. Several studies using microarrays to profile global gene expression [41] and protein signaling [39,40] have revealed an emerging theme in the last few years whereby even so-called “biocompatible” polymers can exert intrinsic biological activity, such as by modulating global gene expression and interfering with important cell signaling cascades. In addition to their nanotoxicology and clinical safety, research on the biological effects of drug/gene delivery carriers may also elucidate potentially unique biological functions that could be used therapeutically [42]. As a result, there is a broad consensus in the nanotechnology community that the clinical importance of biological alterations induced by drug/gene delivery systems in living cells needs to be assessed both in vitro and in vivo.

### 2.1. Physicochemical and Toxicological Properties of Dendrimers

PAMAM dendrimers exhibit some cytotoxicity compared to other polymeric delivery systems. Factors that can induce dendrimer cytotoxicity are dependent on the generation, concentration, period of exposure, surface chemistry, and cell type. Research results show that in comparison to their anionic and neutral counterparts, which are said to have minimal effects on cell viability, positively charged naked PAMAMs can exhibit considerable cytotoxicity (measured in terms of reduced cell viability or cell death) [39,40]. The induced cytotoxicity is primarily due to the interaction between the cationic dendrimer surface and cell membranes, which are negatively charged. The lipid bilayer, which is composed of proteins, phospholipids, and cholesterol, provides a negative charge on the cell membrane surface. The electrostatic interaction between the dendrimer and the cell surface, results in the formation of nanopores, which, in turn, causes damage, leakage of cellular content and cell death [43,44]. PAMAM cytotoxicity has primarily been investigated in vitro using cell lines. It has been suggested that primary cells may be a superior model because cell lines may not accurately reflect the toxicity of PAMAMs in vivo. The cell viability and cytotoxicity of several cationic PAMAM dendrimer generations (G4, G5, G6, and G7), as well as their surface chemistries (cationic, neutral, or anionic), were evaluated using primary aortic vascular smooth muscle cells. Results showed that cytotoxicity was dose-dependent for cationic G5 and increased with subsequent cationic PAMAM dendrimer generations. Neutral and anionic PAMAMs had no significant impact on cell viability in any of the generations tested [40]. Thus, the physicochemical characteristics of PAMAM influence the overall toxicity it causes in primary cells.

Due to these cytotoxic characteristics, cationic polymers such as PAMAMs are being evaluated for both in vitro and in vivo anticancer applications. Additionally, cationic surface chemistry plays a significant role in mediating the toxicological/biological effects of PAMAMs in vivo [42,45]. A recent study investigating the in vivo nanotoxicological effects of the cationic G6 PAMAM in the heart found that prolonged intraperitoneal treatment with this PAMAM significantly hampered the recovery of cardiac function after ischemia–reperfusion injury in rats [46]. Increased cardiac damage markers were found after isolated hearts were exposed to cationic G6 PAMAM [47], most likely indicating PAMAM-mediated myocardial injury and cell death. An in vitro cytotoxicity study of PAMAM in two cancer cell lines (SW480 and HeLa) showed that the G4 PAMAM was the least toxic, and G6 PAMAM was the most toxic, whereas SW480 showed a greater sensitivity to G5 PAMAM. In addition, proinflammatory activities were found to be related to dendrimer generations, i.e., G3 > G2 > G1 > G0 [48]. An in vivo study using a mouse model showed that G5 PAMAM can cause acute lung failure when administered via the intranasal route. The binding of the PAMAM dendrimer to the angiotensin-converting enzyme 2 was shown to be the mechanism of this negative effect, downregulating its function and expression in lung tissue. [49]. Furthermore, G4 PAMAM demonstrated the ability to penetrate neurons after intraventricular injection but did not induce apoptosis at submicromolar concentrations [50].

Toxicity studies on PAMAM dendrimers in vitro and in vivo revealed that amine-terminated PAMAM dendrimers are more toxic than hydroxyl- or carboxylic-acid-terminated PAMAM dendrimers, as evidenced by a study using zebrafish embryos [51]. Surface modifications with biocompatible compounds such as PEG and pyrrolidine and targeting ligands such as folate can reduce the toxicity levels of dendrimers due to a de-crease in the cationic dendrimer charge [43]. Interestingly, the complexation of nucleic acids and/or chemotherapeutic drugs may also significantly reduce dendrimer toxicity. This is possible due to the electrostatic interaction between the therapeutic agent and dendrimer, which reduces the positive charges on the surface of the dendrimer, thereby resulting in a reduction in associated toxicities [44]. Furthermore, its interaction with various biomolecules can be significantly influenced by its zeta potential, i.e., a change in zeta potential was closely correlated with inherent cytotoxicity [52].

### 2.2. Dendrimers as Emerging Nanocarrier Modifiers in Cancer Therapy

Cancer remains a deadly disease with high mortality rates. Owing to its complexity, cancer is increasingly difficult to control. The complexity of cancer is related to the biological diversity of tumors (tumor heterogeneity), the rapid clonal evolution displayed by cancer stem cells, and the development of cancer along several canonical routes [53,54]. Conventional cancer treatments such as surgery, radiation, and chemotherapy are effective methods of treating cancer to some extent, but they are accompanied by detrimental side effects in healthy tissues [55]. These conventional therapeutic approaches are less effective due to the pathophysiology of tumors and aberrant blood vessels. The surface of a dendrimer has multiple functional groups that can be utilized to conjugate a wide range of molecules, including drugs, genetic materials, cell-penetrating peptides and targeting moieties [56,57]. Dendrimers may also be capable of encapsulating these molecules within their dendrimer cavities [58,59]. Due to their great biocompatibility and proven pharmacokinetic characteristics, dendrimers have also been considered for applications in anticancer therapy [60]. They can be used to deliver targeted therapy in all types of cancers because they have a high drug-loading capacity. Dendrimers have been shown to improve the half-lives of drugs, improve stability, and lower immunogenicity [61,62].

Drug molecules can be loaded into the core and/or attached to the functional groups on a dendrimer’s terminal [63] by the processes of encapsulation or complexation. It was reported that cisplatin encapsulated within PAMAM dendrimers showed increased accumulation at the tumor site, as well as decreased cisplatin toxicity [64]. Due to their unique structural characteristics, they also facilitate a sustained release of the drug. The phenomenon of controlled pH-responsive delivery was shown for doxorubicin (DOX)-loaded multifunctional G5-PAMAM dendrimers. The same authors also reported folate-targeted delivery to folate-positive KB cells [65]. Dendrigraft poly-L-lysine (DGL) is another novel polymer in biomedical applications, as it possesses the favorable characteristics of both poly-L-lysine and dendrimers, which have numerous applications in diagnostics, drug and gene delivery, and photodynamic therapy [66,67]. Dendritic polymer-based NPs can be used to deliver chemotherapeutic drugs, such as cisplatin, methotrexate, and camptothecin, with improved accuracy [68]. Due to the high degree of branching and high molecular weights of dendritic polymers, which enable them to be multivalent by nature, the increased permeability of the tumor tissues causes the polymers to accumulate specifically at the tumor site.

Dendritic polymers are available in several forms, including hyperbranched polymers, dendronized polymers, perfect dendrimers, and dendrons. In biomedical applications, PAMAM dendrimers, especially G5 dendrimers, are widely used as scaffolds [35,69]. Poly(propyleneimine) (PPI) dendritic polymers contain many cationic amine groups that may be cytotoxic. DOX and methotrexate have also been codelivered to breast cancer (MCF-7) cells using dendritic magnetic NPs [70].

### 2.3. Passive Targeting for Cancer Therapy

A tumor displays defective blood circulation, which, if exploited, can enable therapeutics to accumulate at the tumor site. Passive targeting can be achieved using dendrimers, as they are capable of directly entering the tumor cells. Due to the abnormal lymphatic drainage and blood supply of cancer tissue, dendrimers may concentrate at the site of the tumor. This could also lead to dendrimer opsonization, as they may be absorbed by nearby macrophages, resulting in non-specific distribution in healthy organs [71]. Dendrimers use the body’s biological mechanisms, such as the enhanced retention system (ERS) or enhanced permeation system (EPS), commonly referred to as the enhanced permeability and retention (EPR) effect, for passive targeting. It has been shown that the terminal functional groups of dendrimers have a high binding affinity with plasma proteins and biomolecules, which can improve the circulation time of the cargo in the blood stream. The tumor microenvironment enables activation or release of therapeutic molecules from the delivery vehicle. Huynh et al (2021) successfully synthesized and dendrimer-functionalized an NP to deliver the p53 tumor suppressor gene, which was administered locally at the tumor site. Results showed an improved antiproliferative effect compared to the free naked gene [72]. It was also demonstrated that dendrimers of the 3.5 generation loaded with cisplatin resulted in considerable accumulation in breast cancer cells [53].

Various types of dendrimers, such as poly(etherhydroxylamine), poly(amidoamine) (PAMAM), poly(ester-amine), poly(propylene imine), and polyglycerol, are favored as novel therapeutic carriers for cancer treatment [73]. Cationic dendrimers, including PAMAM-NH4 (G0-G4), can permeate the biological membrane via endocytosis and the paracellular pathway. When polyethylene glycol (PEG) is added to dendrimers, the resulting effect is an increase in molecular size, as well as a significantly improved circulation time. Furthermore, it can increase the water solubility of dendrimers and ultimately improve their accumulation at the tumor site [74].

### 2.4. Receptor-Targeted Drug Delivery for Cancer Therapy

Passive targeting has its own limitations, such poor targetability, leading to reduced drug doses reaching the cancer tissue and in the development of multidrug-resistant (MDR) malignancies [75]. To maintain the ongoing growth of a malignant mass, cancer cells have the capacity to overexpress several receptors such as folate receptors [76]. Dendrimers conjugated to folate ligands are able to bind with these folate receptors and to bring about folate-receptor-mediated endocytosis. To this end, anticancer drugs such as methotrexate, cisplatin, doxorubicin, etc., could be covalently linked to folate-modified dendrimers for targeted drug or gene delivery [77]. Different ligands that target breast cancer cells can be conjugated to the terminal groups of dendrimers. Furthermore, dendrimers have been conjugated to ligands such as biotin, monoclonal antibodies, amino acids, and carbohydrates for targeted delivery [53,64]. G5 PAMAM dendrimers have demonstrated to achieve successful folate-receptor-targeted delivery of doxorubicin [78], whereas folate coupled to polypropylene imine (PPI) dendrimers were reported to exhibit successful delivery of 5-fluorouracil and fluorescein isothiocyanate (FITC) to breast cancer cells [79].

G5 PAMAM dendrimers coupled to an anti-HER2 monoclonal antibody demonstrated an ability to target HER2-positive cell lines [80]. HER2 receptors are prominent targets in cancer immunotherapy. The overexpression of this protein is observed in a variety of tumor cells, especially in breast cancer cells [81]. In another study using G5 PAMAM dendrimers, methotrexate, an anticancer drug, was used in place of the anti-HER2 monoclonal antibody to inhibit dihydrofolate reductase from converting dihydrofloic acid into tetrahydro folic acid. Breast cancer cells that are HER2-positive are known to be more aggressive than HER2-negative cells [82]. Trastuzumab is a monoclonal antibody approved by the Food and Drug Administration (FDA) for treatment of HER2-positive breast cancer. Trastuzumab treats antibody-dependent cellular toxicity and inhibits HER2-positive receptor downstream signaling [83]. In addition, numerous chemotherapeutic drugs, including taxanes and anthracyclines, may be used in combination with trastuzumab. For instance, Trastuzumab and docetaxel (DTX) have demonstrated a synergistic impact for effective therapy [84].

**Figure 2:**
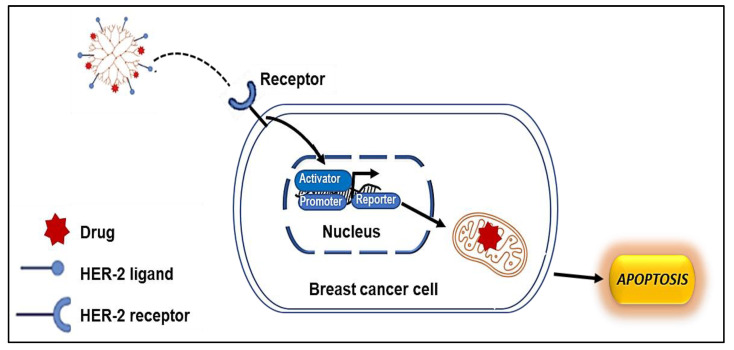
The cellular uptake mechanism of ligand-modified nanocarriers: the ligand-modified nanocarriers enter the cells through the primary endosome after binding to the membrane receptor, followed by the formation of an acidified endosome; later, the fusion of lysosomes ensures the enzymatic degradation of the nanoparticles.

Figure 2 provides a schematic representation of the cellular uptake of a dendrimer-modified NP to specifically target the HER2 receptor in breast cancer cells.

**Figure 3:**
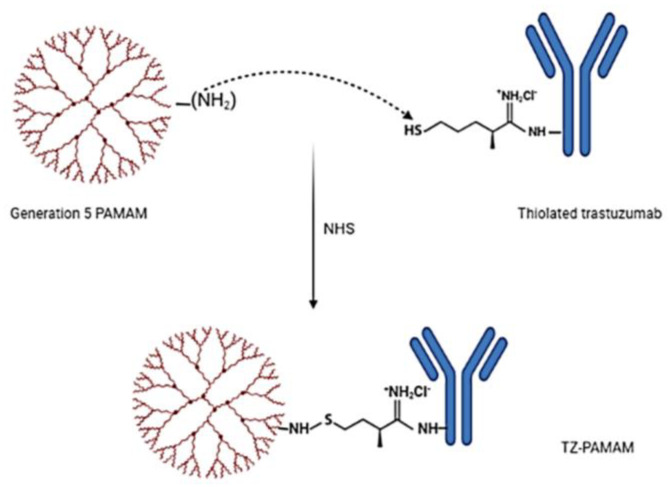
Schematic diagram illustrating the synthesis of trastuzumab (TZ)-grafted G5 poly(amido) amine (PAMAM) dendrimers (created with BioRender.com, accessed 6 December 2022).

Typically, to target cancer cells, trastuzumab is conjugated by covalent bonding along the surface of the dendrimers [85]. Dendrimers conjugated to trastuzumab enhance the delivery of DTX to HER2-positive breast cancer cells. FITC-tagged dendrimers were synthesized for imaging by reacting the primary amine group of the dendrimer with FITC isothiocyanate groups [86]. A schematic representation of the synthesis of trastuzumab-grafted PAMAM dendrimers is provided in Figure 3.

The anticancer efficacy of DTX-loaded plain dendrimers (Dend-DTX, 241.7 3.8 g/mL) and trastuzumab dendrimers (TZ-Dend-DTX, 159.5 5.4 g/mL) was evaluated in breast cancer cell lines (MDA-MB-453 and MDA-MB-231). Dend-DTX released 71.84% of the drug in 24 h and 93.5% in 48 h. A 58.6% release of DTX was achieved by TZ-Dend-DTX after 24 h, with a 73.9% release after 48 h. Therefore, TZ-Dend-DTX demonstrated a better and more regulated release of the drug than Dend-DTX. However, it was observed that the TZ-Dend-DTX combination was 3.57 times more cytotoxic than Dend-DTX. Furthermore, generation 4 PAMAM dendrimer radioimmunoconjugates showed greater toxicity against HER2-positive breast cancer cells [59]. The metastasis noted in breast cancer cells is caused by the expression pattern of the CXC family of chemokines, notably chemokine receptor-4 (CXCR4) [87], which is not found in healthy breast epithelial cells but only in malignant cells. Owing to the presence of this chemokine receptor, cancers cells can spread to various tissues, where stromal fibroblasts express the CXC motif chemokine receptor ligand 12 (CXCL12) [88]. Dendrimers coupled to a ligand directed to the CXCR4 receptors can be used for receptor-targeted delivery of therapeutics. A cyclic pentapeptide known as FC131 (cyclo-D-Tyr-Arg-Arg-L-3-(2-naphthyl)alanine-Gly) demonstrated excellent efficacy as a CXCR4 antagonist [89]. Furthermore, a targeting ligand known as LFC131, a linear form of the FC131 peptide, was used to deliver DOX. Dendrimers were used to encapsulate this pentapeptide to improve it delivery efficacy [90,91]. This resulted in the formulation of a DOX-encapsulated, LFC131-conjugated dendrimer to target the CXCR4 chemokine receptor and to prevent breast cancer from spreading to other organs [92].

Dendrimer size is frequently increased for prolonged blood circulation; however, this comes with drawbacks, such as difficulty in synthesis and the risk of toxicity. Typically, the process of PEGylation has been known to overcome these issues. Hence, to increase circulation time and concentration at the tumor location, dendrimers have been coupled with PEG chains. The extra-peptide linker Gly-Phe-Leu-Gly (GFLG) was reported to bind DOX to the mPEGylated peptide dendrimer at the periphery [93]. The cathepsin B enzyme, which is overexpressed in tumor cells, is known to be sensitive to this linker. These self-assembling NPs have very strong antitumor activity, with PEGylation further diminishing the toxicity induced by DOX. To treat cancers in general, peptide dendrimers are chosen due to their biodegradability and biocompatibility [94].

## 3. Dendrimers and Inorganic Nanoparticles

As mentioned above, dendrimers, especially the PAMAM dendrimers, have been commonly used as drug or gene delivery systems on their own. Anticancer studies feature predominantly due to the multifunctional surface of these dendrimers. Cancer therapy has been applied to lung, liver, breast, cervical, ovarian, glioma, gastric, thyroid, head and neck, and colon cancers, mostly using G4 and G5 dendrimers [95]. Although many novel dendrimeric delivery systems have been formulated over the years, their clinical translation has been limited. Hence, further investigations with the aim of optimizing these interesting polymers are crucial. The use of dendrimers to functionalize inorganic NPs is gaining momentum, with the potential synergism of such dendritic NPs and their therapeutic cargo representing an attractive prospect.

Inorganic NPs have not been as widely used as their organic counterparts or polymeric systems. For the purposes of this review, we will not concentrate on the carbon-based NPs, although they can be classed among inorganic NPs. Overall, inorganic NPs are regarded as having low toxicity, biocompatibility, and stability, especially in terms of storage, availability, ease of synthesis, protection of their therapeutic cargo, and the ability to be modified for conjugation of biomolecules or for cell-specific targeting [24]. Hence, there has been interest, especially over the last decade, in their use in nanomedicine.

Among the many inorganic NPs, gold (Au), platinum (Pt), silver (Ag), and selenium (Se), have been the most popular in biomedicine due to their favorable properties [96], which imbue them with theranostic capabilities. With respect to modification of these NPs, apart from PEG, chitosan, poly-L-lysine, and poly(lactide-co-glycolide), the growing interest in the use of dendrimers has been observed in recent years. Table 1 provide a summary of some interesting dendrimer modifications to inorganic NPs since 2015 that were used for gene or drug delivery. Some of these are NPs are discussed in detail later in this review.

### 3.1. Gold Nanoparticles

**Figure 4:**
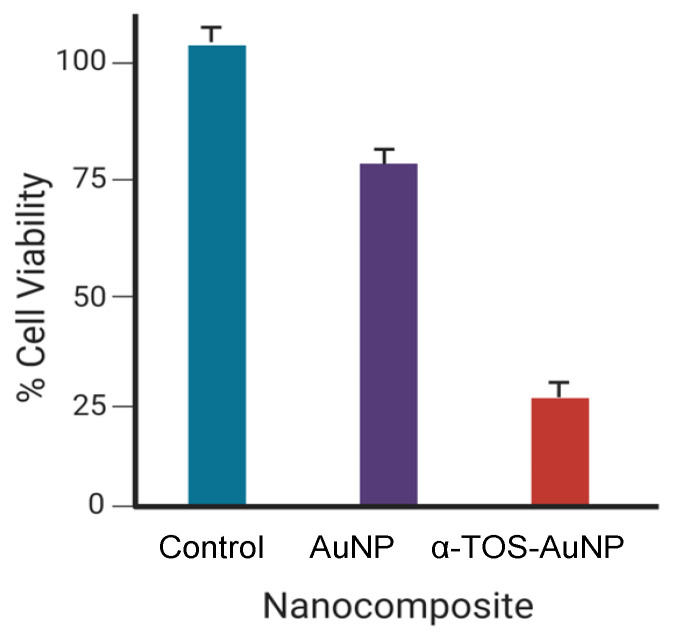
Cell viability graph of α-TOS-AuNP-treated cells compared to unfunctionalized AuNPs using a concentration of 50 μM (Created with BioRender.com, accessed 6 December 2022).

Gold NPs (AuNPs) can be produced in various forms, such as nanospheres, nanorods, nanostars, nanoshells, and nanocages. To date, they have been the most effective and valuable inorganically based NPs, owing to their characteristics such as ease of synthesis, improved biochemical constancy, and exceptional optical properties. As a result, they have received significant technological and scientific interest. These unique characteristics of AuNPs identify them as a promising vehicle for the detection and treatment of diseases including cancer. AuNPs have been shown to have low cytotoxicity in vitro [103]. Furthermore, AuNPs have an affinity for amine and thiol functionalities, which allows them to be easily functionalized via Au–N or Au–S conjugation with targeting moieties, polymers, and certain therapeutic biomolecules [104]. Although AuNPs are used in a variety of medical applications, it is crucial to examine their cytotoxicity both in vitro and in vivo. PAMAM dendrimer-encapsulated AuNPs modified with folic acid (FA) and FITC were investigated in cancer treatment and imaging. At a concentration of 50 nM, the fluorescence of the untargeted dendrimer nanocarriers was lower than that of the folic-acid-modified dendrimer nanocarriers [105]. Acetylated dendrimer nanocarriers loaded with AuNPs were investigated for cancer cell imaging and cell viability, which showed that higher concentrations of AuNPs accelerated the resistance of cancer cells relative to lower concentrations. Acetylation of dendrimers provides good biocompatibility to the cells but has no effect on the cell cycle or morphology according to further flow cytometric studies conducted by the authors [100]. AuNPs functionalized with PAMAM dendrimers coupled with enzyme-linked aptamer and prostate-specific antigen (PSA) were formulated to create an immunosensor for the detection of prostate cancer cells. The conductivity of dendrimers containing PSA (27 μA) was found to be higher than that of dendrimers without PSA (14 μA) according to Nyquist plots. The immunosensor displayed good sensitivity, repeatability, and stability against prostate cancer cells [106]. AuNPs modified with hyaluronic acid and PAMAM dendrimers and carrying the recombinant methioninase (rMETase) gene were used for the prevention of gastric cancer. It was shown that for tumor growth, a 50 mm^3^ tumor initially grew to 250 mm^3^ in 25 days when using a dendrimer carrier with the rMETase gene, as opposed to 400 mm^3^ in 25 days when using a dendrimer nanocarrier without the rMETase gene [5]. The size of an NP is affected by modification with polymers. In a recent study using PAMAM-modified AuNPs for gene delivery, that the naked AuNPs were approximately 65 nm, whereas the PAMAM-modified AuNPs were approximately 100 nm. However, a size reduction was noted upon further modification with the targeting ligand, folate, to 77 nm [36], which could be due to the interaction of the PAMAM chains with the folic acid, giving rise to a more compact NP. AuNPs modified with PAMAM dendrimers were used to encapsulate α-tocopheryl succinate (α-TOS), a vitamin E derivative that can cause apoptosis in cancer cells by obstructing the cell cycle and disrupting the signals between the cancer cells during tumor growth. The cell viability was reduced to 25% as a result of α-TOS (50 μM) being present in the nanocarrier [107]. A graphical representation of this result is provided in Figure 4.

**Figure 5:**
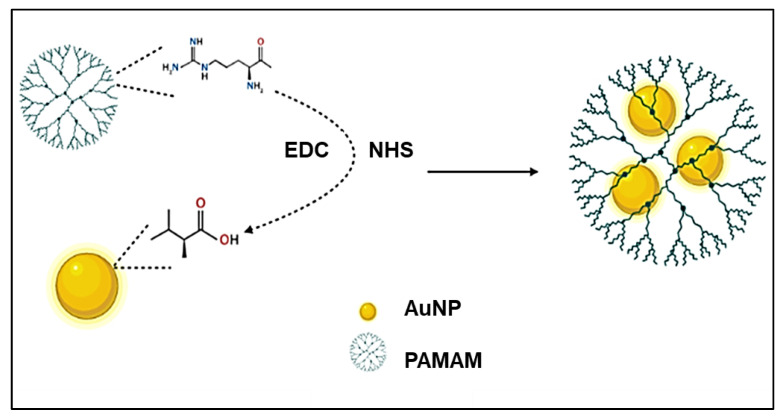
Schematic illustration showing Au–PAMAM NPs synthesized using carbodiimide chemistry (Created with BioRender.com, accessed 6 December 2022).

AuNPs conjugated to PAMAM using carbodiimide chemistry were formulated to crosslink PAMAM dendrimers to AuNPs for non-viral transfection purposes, a schematic representation of which is provided in Figure 5. This chemistry has been widely used, especially in amino acid coupling, and has been shown to enhance colloidal stability and DNA condensation [108]. It was reported that increasing the amine-to-carboxyl ratio during the conjugation of PAMAM onto AuNPs provided the best nanocarrier in terms of colloidal stability and in vitro transfection efficiency. Owing to their commercial availability, ease of synthesis and scaling up, high yield, high transfection efficiency, and minimal cytotoxicity, Au–PAMAM conjugates are promising candidates for non-viral gene delivery [109].

Zwitterion-functionalized, dendrimer-entrapped AuNPs (Au DENPs) were used in a serum-enhanced gene delivery strategy for suppression of cancer cell metastasis in vitro. The AuNPs were encapsulated in G5 PAMAM dendrimers and decorated with zwitterion carboxybetaine acrylamide and the lysosome-targeting chemical morpholine (Mor). Successful transgene expression was noted in cancer cells using both Mor-modified and Mor-free Au DENPs in media with or without serum. The gene delivery efficiency of Mor-modified Au DENPs and the Mor-free Au DENPs in the serum-containing medium were 1.4 and 1.7 times greater than the corresponding NP in the serum-free medium, respectively, due to the antifouling characteristic expressed by the linked carboxybetaine acrylamide zwitterion [110].

### 3.2. Selenium Nanoparticles

**Figure 6:**
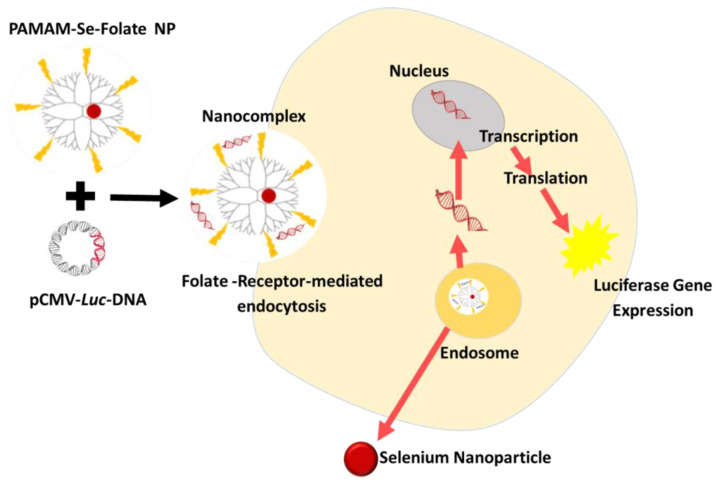
A representation of the delivery and transgene expression of pCMV*-Luc* DNA using folate- targeted, PAMAM-functionalized selenium nanoparticles. Reproduced from [34], MDPI, 2020.

Selenium nanoparticles (SeNPs) have attracted considerable attention over the past five years as potential nanocarriers for the delivery of drugs and genes. However, the poor stability of conventional SeNPs compromises their physicochemical properties and impairs their efficacy. These promising anticancer NPs have undergone numerous improvements to increase their stability and biocompatibility [111]. SeNPs have been coated with various naturally occurring polysaccharides [112,113,114,115], as well as polymers such as poly-L-lysine [24], PEG [116], and chitosan [8,117,118]. The use of dendrimers to functionalize SeNPs has not been fully explored, with only a few studies reporting dendrimer-functionalized SeNPs in gene or drug delivery [34,58]. Pillay et al. (2020) showed enhanced folate-targeted transgene expression in cervical and breast cancer cells in vitro using PAMAM-functionalized SeNPs (Figure 6) [34]. Functionalized SeNPs can deliver genes to the target site, provide antigens for active immunization, and deliver drugs for anticancer treatments. SeNPs are also potential drug nanocarriers, with several indicators pointing to their viability as a reliability. SeNPs functionalized with PAMAM dendrimers were used to concurrently deliver siRNA and cisplatin to A549/DDP cells. This treatment induced cell apoptosis via the PI3K/Akt/mTOR and MAPK/ERK pathways [58]. In a nude mouse model, PAMAM-SeNPs were shown to successfully deliver siRNA and cisplatin to tumor tissue without causing any systemic damage to normal tissue [119].

The main barrier to the use of SeNPs is in the correlation of selenoprotein levels, which is essential for determining the actual pharmacodynamics of therapies and the function of these proteins in generating the apparent protective effect [120].

### 3.3. Silver Nanoparticles

Silver nanoparticles (AgNPs) have been popular due to their conductivity and especially their antibacterial activity. However, they do end aggregate into larger clusters, which reduces their efficiency as a nanocarrier. Biocompatible and biodegradable polymers can be employed as suitable stabilizers for AgNPs [121]. Although there have been very few reports on dendrimer-modified AgNPs in cancer therapy, two recent studies employed AgNPs modified with carbosilane dendrons with and without PEG for the delivery of siRNA. Both studies reported favorable cellular uptake and good anticancer activity [122,123]. Ag has also been popular in the formation of bimetallic NPs, especially with Au. Dendrimer-functionalized Ag–Au NPs have been utilized in drug delivery [100], as indicated in Table 1. Graphene PAMAM dendrimer-functionalized AgNPs have also been employed for the detection of methimazole, a drug used to treat hyperthyroidism [124]. Overall, the use of AgNPs has been limited in cancer therapy, largely due to adverse toxicities, which must be overcome if they are to be considered suitable inorganic NPs that can be modified with dendrimers.

### 3.4. Bimetallic Nanoparticles

Bimetallic dendrimer-encapsulated nanoparticles (DENs) have drawn significant scientific interest due to their promising characteristics, novel biomedical application, and other cutting-edge “nano-” science and technology fields. Some significant applications for bimetallic DENs have been reported, such as gene and drug delivery systems, and research interests have already evolved well beyond the development of a synthetic approach to design dendrimer-functionalized NPs. Bimetallic DENs provide unique characteristics over other nanocarrier systems. As early as 2010, Weir and coworkers reported on Au–Pd DENs with Au as the core and Pd as the shell modified with PAMAM dendrimers. [125]. Most bimetallic DENs have been used for catalytic purposes. However, a recent study reported on the delivery of DOX using Au–Pd nanodendrites. The authors showed that the DOX-containing Au–Pd NPs inhibited breast cancer cell proliferation, together with greater DOX release at pH 4.5 and 5.5 compared to that at pH of 7.4 (Figure 5) [126]. Bimetallic DENs can be used in bioimaging; bimetallic DENs containing a Au core are being explored, owing to the chemiluminescent activity promoted by Au [127]. It was also demonstrated that curcumin-conjugated bimetallic DENs can be selectively taken up by microglial cells in the brain, suggesting that they may be beneficial in the treatment of brain cancer [128]. Additionally, recent research has demonstrated that bimetallic DENs are capable of serving as miRNA carriers [23,129,130,131]. Intravenously injected Au–Pd DENs were also able to deliver miRNA to EGFR-expressing cancer tissues [132]. Moreover, Mg–Fe DENs were shown to effectively target tumors and the kidneys and could be used to treat cancer [133]. Spherical bimetallic NPs (SBNs) also have considerable potential for gene delivery [134]. SBNs often consist of a AuNP core with a different metal as a shell. Although SBNs have been proposed to overcome limitations, such as cytotoxicity and low cellular uptake efficiencies, they often become trapped in endosomes. The strategy of combining dendrimers with SBNs can bypass some of these limitations, as these NPs may induce a proton sponge effect and thus escape from the endosomes [135]. Tianyu et al. (2016) produced Se-shell–Pt-core coordination dendrimers that demonstrated anticancer activity without the conjugation of drugs in vivo in cancer tissue with no adverse effects on normal tissue [136].

Bimetallic DEN-mediated gene or drug delivery appears to be a promising therapeutic strategy in the battle against diseases, including cancer.

### 3.5. Toxicity of Inorganic Nanoparticles

Inorganic NPs vary in their properties and therefore their level of toxicity, if any. AuNPs, the most utilized NPs, have been used in many preclinical studies and have been shown to have little or no toxicity [104]. Furthermore, a previous study showed that a high dose of 2,7 g Au/kg injected into the tail vein of mice produced no adverse hematological or biochemical toxicity [137]. A study by Lin et al. (2018) showed the AuNPs synthesized through diatrozic acid linking using G5 PAMAM as a scaffold to encase the AuNPs did not induce cytotoxicity in HeLa cells or in normal healthy cells in mice, even after conjugation to FA and FITC [138]. In vitro studies using PAMAM-functionalized AuNPs also showed no significant toxicity in tested mammalian cell lines, even after conjugation with DNA, siRNA, or mRNA [35,36,38]. These studies all further demonstrate the biocompatibility and stability of AuNPs, supporting their use in nanomedicine, as well as their potential for polymeric modification. However, it is important to note that the size and concentration of AuNPs are crucial in determining their toxicity level, with in vitro optimizations and modifications playing a significant role.

Selenium (Se) is an essential micronutrient and is known to have therapeutic properties. However, in high concentrations, Se can be toxic to the body and should not exceed a daily intake of 60 μg for men and 53 μg for women [139]. These parameters are important when designing SeNPs for gene or drug delivery. Again, the size and concentration of the SeNPs are crucial. However, SeNPs are the least toxic of the selenospecies and may be superior to metal NPs due to their excellent biocompatibility and degradability in vivo [111]. In a recent study, PAMAMs were reported to induce greater cytotoxicity than SeNPs modified with PAMAM. The authors attributed this phenomenon to the enhanced stabilization and electroneutrality arising from the inclusion of SeNPs, which prevented any membrane phospholipid damage that would cause toxicity [34]. Hence, similar to AuNPs, SeNPs can be considered for applications in nanomedicine.

As previously mentioned, the toxicity and poor stability associated with AgNPs has prevented their continued use in biomedicine. AgNPs and dendrimer-modified AgNPs have been reported to display cytotoxic and hemolytic activity at high concentrations, which excludes their intravenous use [122]. An in vitro study using several mammalian cell lines showed that AgNPs on their own exhibited cell-specific cytotoxicity, with the highest rate of cell death (around 80%) occurring in HepG2 (liver carcinoma) cells at concentrations of 15 and 25 μg [121]. It has been proposed that these challenges can be overcome using suitable modifications, such as polymer coating, and by optimizing the shape and size of the AgNPs [123,140]. Bimetallic NPs have combined properties of two metals and can therefor exhibit similar toxicity levels. This can also depend on which metal resides in the core and which metal forms the shell.

Overall, inorganic NPs can induce toxicities to varying extents depending on the concentrations employed in the study. On the positive side, polymeric functionalization has been shown to provide some advantage to NPs in terms of stability and toxicity. The use of green synthesis of NPs may also contribute to overcoming their cellular toxicity and even reducing the impact of the environment. Greater efforts are needed both in vitro and in vivo to overcome these issues in order to move therapeutics using inorganic NPs from the laboratory to the clinic setting. Hence, researchers need to continually strive to formulate solutions that can reduce inorganic NP cytotoxicity while retaining their favorable properties.

## 4. Conclusions and Future Prospects

Research conducted in the last seven years has shown that inorganic and especially metal-based NPs have the potential to overcome limitations caused by drug resistance in cancer treatment. Au, Ag, and Se NPs, in particular, have been shown to exhibit positive cellular interactions with biomolecules on the cellular surface and within cells. Dendrimers on their own have been used as drug delivery systems but have yet to realize their full potential. An interesting application using dendrimers is to employ them as modifying polymers of inorganic or organic NPs. This functionalization improves the stability of NPs and imbues them with multiple binding sites on their surface to enable the conjugation of various ligands and therapeutic molecules such as monoclonal antibodies, peptide chains, and plasmids. Moreover, the hydrophilic nature of dendrimers allows for the encapsulation of drug molecules and other therapeutic biomolecules. Based on their unique physicochemical properties, several novel drug delivery systems have been developed. The development of multifunctional, highly selective nanocarriers is primarily driven by advances in dendrimer research and the growing demand for combination therapeutic approaches. Research conducted in the past seven years has demonstrated that dendrimer-based delivery systems are becoming more widely used, which will significantly increase the therapeutic indices of currently available cancer treatments, in addition to extending their use to clinics.

However, as with all carriers, it is necessary to thoroughly assess the safety and effectiveness of dendrimers as therapeutic carriers before undertaking in vivo trials. Associated dendrimer toxicities can be circumvented by surface modifications with biocompatible compounds, reducing their cationic nature. Taking this into consideration, the use of dendrimers as an NP modifier can provide a twofold advantage. First, surface modification of NPs can reduce the cationic charge of dendrimers, thereby reducing the associated dendrimer toxicity. Secondly, the presence of a dendrimer on an NP surface allows for stabilization of the nanocomplex and complexation with the therapeutic agent (nucleic acid and/or drug), potentially enhancing their cancer therapeutic potential. Dendrimer-based NPs must also overcome production challenges associated with scaling up because numerous techniques have been typically used over the years and need to be rationalized. Overall, dendrimers and dendrimer-based NPs have paved the way for a number of possible medical applications with the aim of improving and advancing cancer treatment for the foreseeable future. Elucidation of the exact mechanisms involved in these adverse issues will go a long way in combatting in vivo toxicity. Using smart novel designs, the formulation of multifunctional inorganic NPs can be realized for their application in various facets of cancer, including therapy, diagnostics, and imaging.

## Figures and Tables

**Table 1:** Summary of dendrimer-modified inorganic nanoparticles in gene and drug delivery.

Nanoparticle	Dendrimer	Outcomes	Ref
SeNP	PAMAM	Improved compaction and protection of plasmid DNA from enzyme digestion, as well as enhanced transgene expression in cervical cancer cells.	[34]
AuNP	PAMAM	Improved mRNA delivery to MCF-7 breast cancer cells by more than 80%.	[36]
		Safe and efficient delivery of plasmid DNA with improved transgene expression.	[35]
		siRNA-based proof-of-principle study showing significant gene silencing in HeLa-tat-*Luc* cells.	[38]
Fe_3_O_4_/SiO_2_	PAMAM	High drug-loading efficiency (90%) and 95% drug release in vitro.	[97]
AuNP	PEG-PLL Dendrimer	Drug-conjugated dendrimer reduced lung cancer by 95%.	[98]
AuNP	PAMAM	Successfully delivered the METase gene, which inhibited gastric tumor growth.	[99]
Ag–Au	Poly(L-lactide) Dendrimer	Reduced cytotoxicity by 90% and increased drug release by 86%.	[100]
Fe_3_O_4_	PAMAM	Induced significant apoptosis in cervical cancer cells.	[101]
	Dendritic Cs-g-mPEG	Successful codelivery of doxorubicin and methotrexate.	[70]
Pd/AuNP	PAMAM	Successful codelivery of gemcitabine and miR-21 inhibitor in pancreatic cancer cells.	[102]

## Data Availability

Not applicable.

## Abbreviations

Ag	Silver
Akt	Alpha serine/threonine protein kinase
Au	Gold
CXCR4	CXC motif chemokine receptor 4
CXCL12	Chemokine receptor ligand 12
DGL	Dendrigraft poly-lysine
DEN	Dendrimer-encapsulated nanoparticle
DOX	Doxorubicin
DTX	Docetaxel
EGFR	Epidermal growth factor receptor
EPS	Enhanced permeation system
ERK	Extracellular signal-regulated kinases
ERS	Enhanced retention system
FA	Folic acid
FC131	Cyclo-D-Tyr-Arg-Arg-L-3-(2-naphthyl) alanine-Gly
FDA	Food and Drug Administration
FITC	Fluorescein isothiocynate
G	Generation
GFLG	Gly-phe-leu-gly
HER	Human epidermal growth receptor
MAPK	Mitogen-activated protein kinase
MDR	Multidrug resistance
MgFe	Magnesium iron
Mor	Morpholine
MRI	Magnetic resonance imaging
mTOR	Mammalian target of rapamycin
NP	Nanoparticle
PAMAM	Poly(amidoamine)
PEG	Polyethylene glycol
PPI	Polypropylene imine
PR	Progesterone receptor
PSA	Prostate-specific antigen
Pt	Platinum
PTT	Photothermal therapy
PTX	Paclitaxel
PI3K	Plasma membrane-associated lipid kinase
rMETase	Recombinant methioninase
Se	Selenium
TAT	Transactivating transcriptional activator
α-TOS	α-tocopheryl succinate
TZ	Trastuzumab
